# Multifunctional Roles of Betulinic Acid in Cancer Chemoprevention: Spotlight on JAK/STAT, VEGF, EGF/EGFR, TRAIL/TRAIL-R, AKT/mTOR and Non-Coding RNAs in the Inhibition of Carcinogenesis and Metastasis

**DOI:** 10.3390/molecules28010067

**Published:** 2022-12-21

**Authors:** Ammad Ahmad Farooqi, Assiya Turgambayeva, Gulnara Tashenova, Aigul Tulebayeva, Aigul Bazarbayeva, Gulnara Kapanova, Symbat Abzaliyeva

**Affiliations:** 1 Department of Molecular Oncology, Institute of Biomedical and Genetic Engineering (IBGE), Islamabad 54000, Pakistan; 2 Department of Public Health and Management, NJSC “Astana Medical University”, Astana 010000, Kazakhstan; 3 Asfendiyarov Kazakh National Medical University, Almaty 050040, Kazakhstan; 4 Scientific Center of Pediatrics and Pediatric Surgery, Almaty 050060, Kazakhstan; 5 Scientific Center of Anti-Infectious Drugs, 75 al-Faraby Ave, Almaty 050040, Kazakhstan; 6 Al-Farabi Kazakh National University, 71 al-Farabi Ave, Almaty 050040, Kazakhstan

**Keywords:** cancer, apoptosis, JAK/STAT, VEGF/VEGFR, EGF/EGFR, AKT/mTOR, betulinic acid

## Abstract

The pursual of novel anticancer molecules from natural sources has gained worthwhile appreciation, and a significant fraction of conceptual knowledge has revolutionized our understanding about heterogeneous nature of cancer. Betulinic acid has fascinated interdisciplinary researchers due to its tremendous pharmacological properties. Ground-breaking discoveries have unraveled previously unprecedented empirical proof-of-concept about momentous chemopreventive role of betulinic acid against carcinogenesis and metastasis. Deregulation of cell signaling pathways has been reported to play a linchpin role in cancer progression and colonization of metastatically competent cancer cells to the distant organs for the development of secondary tumors. Importantly, betulinic acid has demonstrated unique properties to mechanistically modulate oncogenic transduction cascades. In this mini-review, we have attempted to provide a sophisticated compendium of regulatory role of betulinic acid in cancer chemoprevention. We have partitioned this multi-component review into different sections in which we summarized landmark research-works which highlighted betulinic acid mediated regulation of JAK/STAT, VEGF, EGF/EGFR, TRAIL/TRAIL-R, AKT/mTOR and ubiquitination pathways in the inhibition of cancer. In parallel, betulinic acid mediated regulation of signaling cascades and non-coding RNAs will be critically analyzed in cell culture and animal model studies. Better comprehension of the pharmaceutical features of betulinic acid and mapping of the existing knowledge gaps will be valuable in the translatability of preclinical studies into rationally designed clinical trials.

## 1. Introduction

Pioneering research-works over decades have enabled us to dissect multiple mechanisms which underlie carcinogenesis and metastasis [1,2]. Importantly, continuously evolving cancer biology research and the discoveries of new mechanistic paradigms in the study of metastasis have revealed detailed analysis of the molecular underpinnings of this multi-step dissemination process. Cancer cells from primary tumors display hallmark features and unique ability to metastasize to the distant organs [3,4]. Scientists have witnessed extra-ordinary advancements at a breakneck pace and these breakthroughs have increased the optimism for pharmacological targeting of oncogenic pathways for cancer chemoprevention [5,6,7,8]. In accordance with this approach, identification of bioactive molecules having minimum off-target effects and maximum efficacy for cancer chemoprevention has remained an overarching goal for molecular oncologists and medicinal chemists. Hydroxy pentacyclic triterpene acids have gained limelight due to their tremendous medicinal significance and pharmacological research related to these bioactive molecules has gained remarkable momentum. Therefore, the ongoing resolution of the puzzling mysteries underlying carcinogenesis and metastasis has paved the way for targeting of the oncogenic transduction cascades. The expanding scale and inherent complexity of biological data have provided opportunities for innovative therapeutic interventions for different cancers. Different types of proteins participate in the assembly of signaling complexes. Deregulation of cell signaling pathways played central role in multiple stages of carcinogenesis and metastasis.

Pharmacological properties of betulinic acid have been experimentally validated in different disease models. Betulinic acid has a molecular weight of 456.7. For the structural frameworks of the mini-review, we scrupulously browsed PubMed using miscellaneous keywords, particularly “betulinic acid and cancer”, “betulinic acid and metastasis” and “betulinic acid and signaling”. Different keywords generated 242 retrievals. We shortlisted the most relevant, technically sound research works published in high-quality journals. Seminal research works have substantiated central role of betulinic acid in amelioration of diabetes, obesity and atherosclerosis [9,10,11,12,13,14,15,16,17]. Furthermore, betulinic acid has garnered interest in the context of molecular oncology due to its ability to inhibit and prevent cancer. There are some good mini-reviews about cancer chemopreventive role of betulinic acid against wide variety of cancers [18,19,20,21].

We have subdivided this multi-component review into underlying mechanisms which modulate carcinogenesis and metastasis. We have discussed the most important pathways reported to be regulated by betulinic acid. We have given special attention to betulinic acid mediated regulation of JAK/STAT, VEGF, EGF/EGFR, TRAIL/TRAIL-R, AKT/mTOR and ubiquitination pathways in the inhibition of cancer.

We have selected these pathways due to their mechanistic role in carcinogenesis and metastasis. JAK/STAT, VEGF and EGF/EGFR-mediated pathways are primarily involved in developmental stages but, interestingly, deregulation of these pathways has been widely documented during different stages of cancer. Therefore, we have given special attention to regulation of these pathways by betulinic acid.

Lastly, we will identify the knowledge gaps in our understanding about pharmacological targeting of oncogenic pathways by betulinic acid which will be advantageous in effective transition of betulinic acid from preclinical studies to various phases of clinical trials.

In the upcoming section, we will first provide a summary of regulation of JAK/STAT pathway by betulinic acid.

## 2. Regulation of JAK/STAT Signaling Pathway

Wealth of information gathered through structural biology and receptor pharmacology has allowed researchers to unlock the mechanisms of cytokine receptor activation, signifying that different aspects of cytokine function are highly tunable. Kinases of Janus kinase family (JAK) and transcriptional factors of STAT (signal transducer and activator of transcription) family are composed of a rapid membrane-to-nucleus signal transduction module that centrally drives carcinogenesis [22,23,24,25]. Binding of different cytokines to their native receptors triggers the phosphorylation of receptor-associated JAKs [26,27]. There are different STAT proteins which act as downstream effectors in JAK//STAT signaling, but available evidence highlights betulinic acid mediated regulation of STAT3 proteins.

Over decades of pioneering research, steady growth in the field of MMPs (matrix metalloproteinases) has highlighted their fundamental roles in invasion and metastasis. Betulinic acid reduced the expression of MMP2 and MMP9 while simultaneously upregulated the expression of TIMP2 in 4T1 and MDA-MB-231 cells. Likewise, treatment with betulinic acid considerably reduced the levels of p-STAT3 in MDA-MB-231 and 4T1 cancer cells. Importantly, p-STAT3 has been shown to transcriptionally modulate MMP2, MMP9 and TIMP2. Betulinic acid efficiently suppressed pulmonary metastatic nodules in animal models intravenously injected with 4T1 cancer cells. Betulinic acid inhibited the expression of MMP2, MMP9 and p-STAT3 in 4T1 tumor tissues. Myeloid-derived suppressor cells (MDSCs) expand during cancer and inflammation having potent ability to suppress T-cell responses. Betulinic acid strongly suppressed the infiltration of MDSCs into the tumors and lungs and inhibited colonization of tumor cells to distantly located organs [28]. These clues provided impressive evidence that betulinic acid not only interfered with STAT3-mediated signaling but also downregulated metastasis associated proteins to inhibit the spread of metastatic spread of cancer cells.

The wealth of information has also provided direct pieces of evidence related to STAT3-mediated regulation of myriad of oncogenic networks. Importantly, p-STAT3 has been shown to stimulate the expression of c-Myc, CCND1 and BCL-xL [29]. Overall, inactivation of STAT3 will be significant in the shutting down of STAT3-induced oncogenic networks.

Triterpenes from Helicteres angustifolia (HT) such as oleanic acid, helicteric acid and betulinic acid have previously been reported to be effective against colorectal cancer cells by inhibition of STAT3-mediated upregulation of oncogenic networks [30].

Betulinic acid mediated chemopreventive effects have been analyzed in animal model studies as well. Betulinic acid effectively retarded the growth of the tumor xenografts in BALB/c mice subcutaneously transplanted with KB cells. Moreover, betulinic acid reduced the levels of p-STAT3 in tumor tissues derived from KB cells [31].

Hypoxia has been shown to promote prostate cancer via activation of STAT3 and HIF-1α -driven signaling. Betulinic acid suppressed the binding of STAT3 and HIF-1α to promoter regions of VEGF in hypoxic conditions [32].

**Figure 1:**
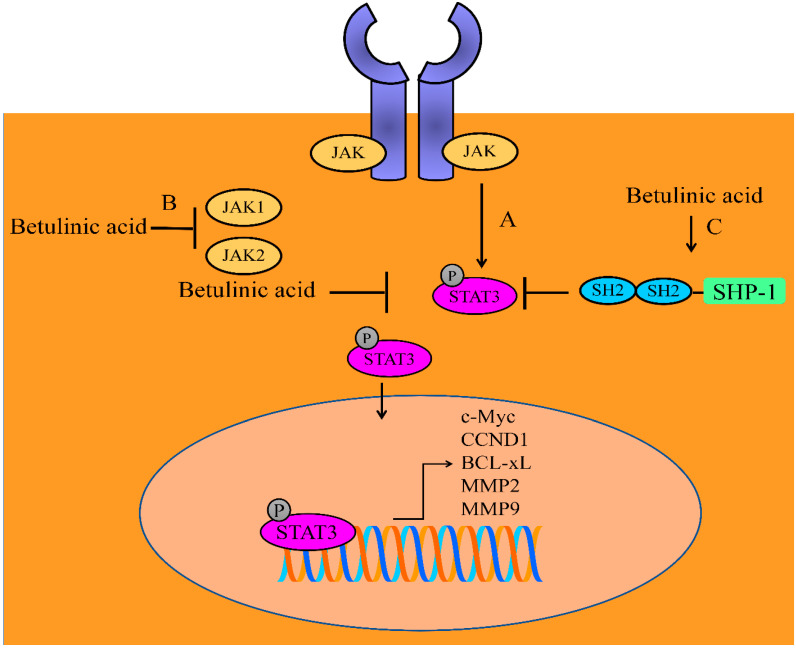
Betulinic acid mediated regulation of JAK/STAT signaling. (A) Janus kinase (JAK)/signal transducer and activator of transcription (STAT) triggered upregulation of target gene network. (B) Betulinic acid inhibited JAK1 and JAK2. (C) Betulinic acid triggered SHP-1 mediated dephosphorylation of STAT3. Abbreviations: CCND1 (Cyclin D1), MMP2/MMP9 (matrix metalloproteinase-2/9), SHP-1 (Src homology region 2 domain-containing phosphatase 1).

Betulinic acid exerted inhibitory effects on the constitutive phosphorylation of JAK1 and JAK2 (Figure 1). Additionally, STAT3 activation, nuclear transportation and transcriptional regulation of gene networks were found to be reduced in betulinic acid-treated multiple myeloma cells. It potentiated the apoptosis inducing effects of thalidomide and bortezomib in MM cells. Betulinic acid induced an increase in the levels of SHP-1 (Figure 1). Importantly, betulinic acid mediated inactivation of STAT3 was noted to be dramatically impaired in SHP-1-silenced cells [33].

It is encouraging to note that betulinic acid remarkably regulates JAK/STAT signaling pathway. However, future studies must converge on the identification of additional regulators of JAK/STAT pathway for cancer chemoprevention. Furthermore, pharmacological targeting of JAK/STAT pathway needs to be tested more comprehensively in different cancer models. In the next section, we will discuss how betulinic acid regulated VEGF pathway. Tumors have significant variability in the patterning and features of angiogenic blood vessels, as well as in their responses to anti-angiogenic therapy. The process of angiogenesis is regulated by pro-angiogenic factors and is now very well recognized as a control switch in tumor development.

## 3. Regulation of VEGF

Angiogenesis is a complex process by which tumors develop new vasculatures to fulfil the core demands of rapidly proliferating cells for the growth of the tumors. Importantly, a significantly characterized member of VEGF (Vascular endothelial growth factors) family is VEGFA (commonly known as VEGF). VEGF ligands bind to and trigger the activation of type III receptor tyrosine kinases. Specificity proteins have been shown to transcriptionally regulate VEGF in different cancers. In this section, we have gathered evidence about betulinic acid mediated inhibition of VEGF.

Specificity proteins have a critical role in the regulation of VEGF. Betulinic acid mediated targeting of specificity proteins is interesting and pharmaceutically valuable. Betulinic acid reduced the levels of Sp1, Sp3 and Sp4 in SK-MEL2 melanoma cells. Essentially, betulinic acid-mediated degradations of Sp proteins resulted in notable suppression in the levels of VEGF in LNCaP cancer cells. Therefore, betulinic acid effectively reduced Sp proteins and VEGF expression in the tumor tissues of xenografted mice [34].

**Figure 2:**
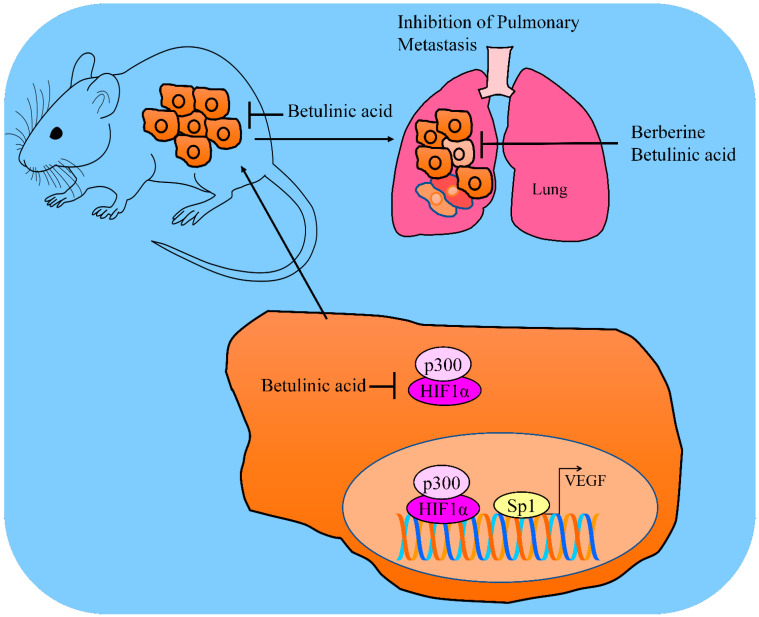
Betulinic acid mediated inhibition of HIF1α and Sp1 mediated upregulation of VEGF. Betulinic acid inhibited the tumorigenesis and metastasis in animal models. Betulinic acid attenuated pulmonary metastatic nodules in mice. Abbreviations: Hypoxia inducible factor (HIF), Vascular endothelial growth factors (VEGF), Histone acetyltransferase p300, Specificity protein (SP).

Combination treatments have also demonstrated scientifically relevant results. Betulinic acid and mithramycin blocked Sp1-mediated upregulation of VEGF in PANC-1 cells (Figure 2). Ectopic overexpression of Sp1 led to resistance against betulinic acid in PANC-1 and BxPC-3 cancer cells [35].

Chidamide (HDAC inhibitor)-mediated p300 over-acetylation suppressed the interactions of p300 with HIF1α. Dissociation of p300 and HIF1α consequently suppressed HIF1α pathway and inhibited HIF1α-mediated transcriptional upregulation of VEGF (Figure 2). Betulinic acid and chidamide combinatorially suppressed tumor growth in mice inoculated with THP1 cells and overexpression of SOD2/HIF1C severely abrogated inhibitory effects [36].

Formulations of natural products exert cancer inhibitory effects and minimum off-target effects. Berberine and betulinic acid in spray dried (SD) mucoadhesive microparticle formulations were prepared and tested for efficacy against animal models orthotopically implanted with A549 cancer cells. Importantly, lung tumor weights were found to be considerably suppressed by berberine and betulinic acid spay-dried formulations. In metastatic lung tumor models, the number of tumor nodules on the surface of medial, peripheral and central lobes were found to be significantly reduced by berberine and betulinic acid. The HIF1α/VEGF pathway was inactivated in the tumor tissues in animal models treated with berberine and betulinic acid [37].

## 4. Regulation of EGFR

Epidermal growth factor receptor (EGFR) has been shown to play critical role in carcinogenesis and metastasis. Following ligand binding, EGFR stimulated downstream cell signaling cascades that influenced uncontrolled proliferation, migration and tumorigenesis. The greater-than-ever expanding knowledge of the spectrum and biology of the mechanisms of EGFR resistance has already propelled the development of more effective therapeutic strategies [38,39,40,41]. In this section, we have summarized the updates related to mechanisms of resistance, evaluation of betulinic acid as a next-generation EGFR inhibitor and approaches to tackle resistance in different cancers.

Betulinic acid and curcumin exerted repressive effects on EGFR levels in 253JB-V and KU7 cancer cells. Levels of EGFR were found to be reduced in Sp1-knockdown or Sp3-knockdown cancer cells. Betulinic acid-dependent and curcumin-dependent downregulation of Sp1, Sp3 and Sp4 effectively suppressed binding of Sp to GC-rich regions within promoter of EGFR [42].

There is gradual development of resistance against first-generation of tyrosine kinase inhibitor therapy in EGFR-mutated patients. Betulinic acid improved resistance against tyrosine kinase inhibitors and induced apoptotic death in lung cancer cells [43].

Nevertheless, certain hints highlighted betulinic acid mediated activation of EGFR/AKT pathway in melanoma cells [44]. These aspects are challenging and future studies related to EGFR activation by betulinic acid must be conducted comprehensively. However, overall, a major fraction of evidence substantiated the role of betulinic acid in the inactivation of EGFR-driven downstream signaling in different cancers.

In the next section, we have provided a summary of regulation of TRAIL pathway by betulinic acid. TRAIL-driven signaling has unique features mainly in context of targeted killing of cancer cells. These exciting aspects make TRAIL pathway an important cascade for cancer inhibition.

## 5. Regulation of TRAIL-Mediated Signaling

There has always been a quest for anticancer agents having minimum off-target and maximum cancer preventive/inhibitory effects. TRAIL (TNF-related apoptosis-inducing ligand)-mediated signals are transmitted by molecular mechanisms that show a high degree of interconnectivity. Binding of TRAIL to death receptors (DR4 or DR5) resulted in receptor trimerization and recruitment of FADD (Fas associated Death Domain protein) through the death domains of DR4 or DR5. Additionally, attachment of pro-caspase-8 to FADD through death effector domains promoted the formation of multi-protein signalosome DISC (Death inducible signaling complex) which activated caspase-8. For the last two decades, an outstanding question within the evolving field of “extrinsically induced apoptosis” has been related to the strategies to target the death receptor arm of the apoptotic pathway to inhibit tumor progression in preclinical models and clinical trials [45,46,47,48,49,50]. Whilst TRAIL-based therapeutics and agonistic death receptor antibodies have classically been heralded as promising anticancer agents, the reality has been more challenging due to loss of cell surface receptors and imbalance of anti- and pro-apoptotic proteins in different cancers.

**Figure 3:**
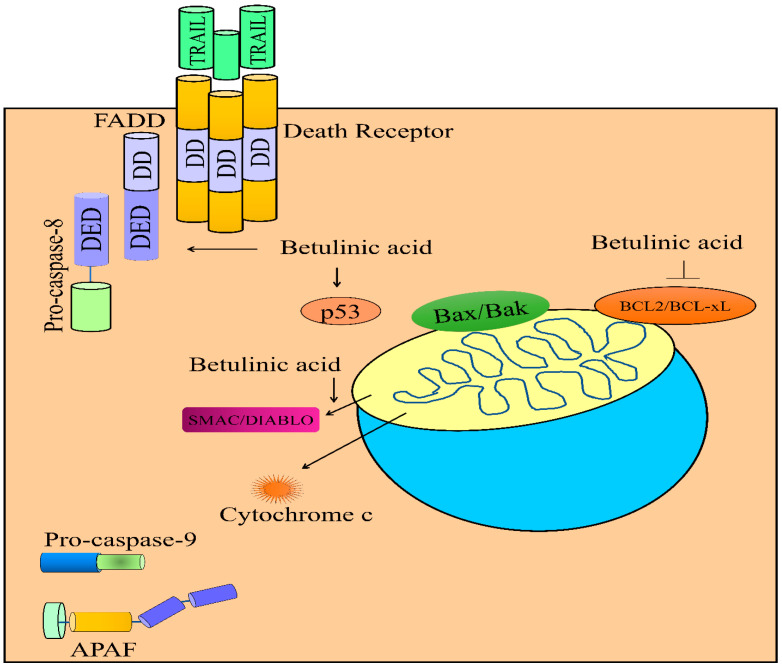
Regulation of TRAIL-mediated apoptosis by betulinic acid. Betulinic acid enhanced the levels of DR4/DR5 and FADD. Betulinic acid activated DISC (death inducible signaling complex). Betulinic acid triggered the release of cytochrome c and SMAC/DIABLO. Betulinic acid increased the levels of p53. Abbreviations: DR4/5 (Death receptor), FADD (Fas-associated death domain protein), DD (Death domain), DED (Death effector domain), APAF-1 (Apoptotic protease activating factor-1), Smac/DIABLO (Second mitochondria-derived activator of caspase/direct inhibitor of apoptosis-binding protein with low pI), MCL1 (Myeloid cell leukemia-1).

Studies have shown that natural products have unique medicinal properties and burgeoning evidence suggested that a loss of cell surface expression of death receptors induced resistance against TRAIL-mediated apoptosis. Therefore, identification of natural products as robust sensitizers of TRAIL pathway is a rapidly evolving field. Betulinic acid independently inhibited clonogenic growth of PLC/PRT/5 and Huh7 cells. Moreover, betulinic acid and TRAIL co-treatments remarkably impaired clonogenic growth of PLC/PRT/5 and Huh7 cells. Betulinic acid and TRAIL combinatorially upregulated p53, DR4, DR5 and FADD levels (Figure 3). Combinatorially, both molecules effectively reduced BCL-2 and MCL-1 and simultaneously enhanced Bad and Bak levels. More importantly, combinatorial treatments exerted stimulatory effects on the levels of p53, DR4, DR5 and FADD in the tumor tissues derived from Huh7 cells [51].

Greater-than-ever knowledge of the role of mitochondria in cell biology has undergone substantial broadening and, intriguingly, scientists uncovered unprecedented roles of mitochondria as key platforms for a plethora of cell signaling pathways. In particular, it was observed that during apoptosis, mitochondria released proteins, such as cytochrome c, SMAC/DIABLO and Omi/HTRA into the cytosol. Bax and Bak permeabilized the outer membranes of mitochondria. Betulinic acid and TRAIL synergistically triggered the release of SMAC/DIABLO and cytochrome c from mitochondria in SHEP neuroblastoma cells. Betulinic acid and TRAIL did not activate apoptotic death in BCL-2 overexpressing SHEP cells [52]. Betulinic acid has also been reported to work efficiently with doxorubicin and potentiate the release of SMAC/DIABLO and cytochrome c from mitochondria in neuroblastoma cells (Figure 3) [53].

Overall, these findings are interesting but need additional research. Upregulation of death receptors on cell surface of TRAIL-resistant cancers is an exciting area of research. Therefore, mechanism-based investigation of betulinic acid mediated increase in the levels DR4 and DR4 will be helpful in the rationalization of combinatorial use of betulinic acid with TRAIL-based therapeutics. There are some outstanding questions related to regulation of TRAIL-mediated tumor suppression by betulinic acid in different cancers. Likewise, TRAIL-mediated inhibition of metastasis in rodent models is also an area of exciting research. Certain clues have been identified, but comprehensive analysis of betulinic acid mediated regulation of TRAIL-driven pathway will be valuable in translatability of true potential of betulinic acid.

## 6. Regulation of Ubiquitination

Skp1-Cullin1-F-box protein (SCF) multi-protein complex played critical roles in carcinogenesis and metastasis by regulation of protein degradation. Betulinic acid and camptothecin prevented the interactions between Skp2 and Skp1. Betulinic acid abrogated Skp2-Skp1 interactions and inhibited Skp2-SCF E3 ligase activity. Betulinic acid evidently reduced Skp2-mediated ubiquitylation of E-cadherin and p27. Betulinic acid caused significant inhibition of primary tumor growth as well as spontaneous lung metastasis without distressing the body weight of the mice. Betulinic acid downregulated Skp2, while simultaneously upregulating E-cadherin and p27 levels in the subcutaneous-xenografted tumor tissues. More importantly, administration of betulinic acid also considerably reduced the size and number of metastatic lung nodules and extended the lifespans of rodent models [54].

The β1 subunit has peptidylglutamyl-peptide hydrolyzing activity, β2 has trypsin-like activity and β5 has chymotrypsin-like activities. Betulinic acid inhibited the accumulation HIF-1α by activation of proteasome-mediated degradation of HIF-1α [55].

For the integrity and maintenance of an intact mitochondrial network, cells have developed highly sophisticated and orchestrated quality control systems that promptly remove superfluous or damaged mitochondria by selective mitochondrial autophagy called mitophagy. In functional mitochondria, PINK1 is imported into the inner mitochondrial membrane, where it is processed and cleaved by different proteases. However, in dysfunctional mitochondria, PINK1 degradation process is halted, which promotes PINK1 accumulation on the mitochondrial outer membrane. Here, PINK1 is activated and it phosphorylates mitochondrially-tethered ubiquitin. Phosphorylated ubiquitin serves as a platform for the recruitment of Parkin. Sequentially, Parkin ubiquitylated different proteins on the MOM to mark mitochondria for targeted degradation. B5G1, a new derivative of betulinic acid significantly upregulated PINK1 in MCF7/ADR (drug-resistant) and HepG2/ADM (drug-resistant) cells. Parkin translocated to the mitochondria, and p-Parkin (Ser65) was upregulated by B5G1 in MCF7/ADR and HepG2/ADM cells. Collectively, these results indicated that B5G1 induced mitochondrial PINK1 upregulation and promoted the recruitment of Parkin and initialized mitophagy. B5G1 potently and remarkably suppressed the growth of HepG2/ADM xenografts. Importantly, B5G1 markedly enhanced PINK1 and p-Parkin levels and activated mitophagy for the clearance of damaged mitochondrial proteins [56].

SUMO1 (Small ubiquitin like modifier 1) increased Sp1 sumoylation and consequently reduced Sp1 levels. Sp1 transcriptionally downregulated PTEN (Phosphatase and tensin homolog). Betulinic acid enhanced SUMO1 and triggered SUMO1 mediated Sp1 sumoylation. Resultantly, Sp1 was unable to repress the expression of PTEN in betulinic acid -treated CAL-27 cells [57].

SENP1, a member of the de-SUMOylation protease family has central role in carcinogenesis. Betulinic acid increased the levels of SUMO-Sp1 by downregulation of SENP1 expression. Sp1 sumoylation induced an increase in the interaction with RNF4, which resulted in Sp1 degradation in a ubiquitination-mediated proteasome-dependent pathway. Betulinic acid reduced Sp1 levels and increased the levels of caspase 3 in the bitransgenic lung tumor rodent model [58].

## 7. Regulation of AKT/mTOR

Seminal studies had shown that AKT/mTOR signaling acted as a central driver of carcinogenesis and metastasis. Therefore, betulinic acid modulation of AKT/mTOR pathway is certainly exciting. Betulinic acid effectively suppressed the levels of p-PI3K, p-AKT and p-mTOR in HepG2 and SMMC-7721 cells. Collectively, these findings indicated that betulinic acid mediated inactivation of PI3K/AKT/mTOR axis resulted in the apoptotic death of HepG2 and SMMC-7721 cells [59].

Betulinic acid induced activation of AMPK and consequently reduced the activation of mTOR. Phosphorylated levels of mTOR were found to be reduced by betulinic acid in PANC-1 and SW1990 cells. mTORC1 phosphorylated p70S6K but betulinic acid interfered with mTORC1-mediated phosphorylation of p70S6K. Moreover, betulinic acid caused shrinkage of the tumor xenografts in mice inoculated with PANC-1 cancer cells [60]. Overall, greater comprehension of the mechanism that leads to targeting of hyperactive AKT/mTOR pathway may provide valuable insights to help design new strategies that will enhance the impact of betulinic acid in cancer chemoprevention.

## 8. Regulation of Non-Coding RNAs

Amazing discovery of non-coding RNAs has not only dramatically reshaped our understanding about various facets of molecular biology and rapidly broadening comprehension of RNA functions and their crucial roles in carcinogenesis and metastasis are unprecedentedly exciting. High-throughput technologies have enabled us to unravel and characterize different types of non-coding RNAs which play central role during carcinogenesis and metastasis. Contemporary studies have highlighted the role of microRNAs and long non-coding RNAs in regulation of multiple steps of carcinogenesis [61,62,63,64,65,66,67]. In this section, we will emphasize the regulation of microRNAs by betulinic acid due to limited evidence about regulation of lncRNAs and circular RNAs.

p66shc, a central redox sensor, is principally localized in mitochondria and regulated by p53. Diethylnitrosamine (DEN) and CCl4 caused considerable reduction in p66shc expression, as well as phosphorylation in the liver tissues of mice. Moreover, p66shc protein levels and phosphorylation were upregulated by betulinic acid in HCC cells as well as in the liver tissues of mice treated with diethylnitrosamine and CCl4. Injections of p66shc shRNA evidently enhanced the number and size of the tumors in D/C + BA-treated rodent models. Additionally, injections of p66shc shRNA in mice markedly enhanced tumor growth and concurrently reduced the rate of apoptosis in cells. p53 transcriptionally upregulated p66shc and miR-21 in cancer cells. Studies have shown that miR-21 targeted SOD2 in cancer cells. Betulinic acid stimulated the expression levels of miR-21 in HCC cells and liver tissues of mice treated with diethylnitrosamine and CCl4 [68]. Collectively, p53 increased miR-21 levels and inhibited SOD2 levels, leading to significant increase in the accumulation of ROS levels and apoptotic cell death.

Betulinic acid reduced miRNA-27a levels and induced ZBTB10 in MDA-MB-453 and BT474 cancer cells. miR-27a directly targeted ZBTB10 and stimulated the levels of Sp1, Sp3 and Sp4. Tumor volumes and weights were inhibited significantly. Moreover, betulinic acid exerted repressive effects on the levels of Sp1, Sp3 and Sp4 in tumor tissues of mice xenografted with BT474 cancer cells [69]. Likewise, in colon cancer cells, betulinic acid blocked miRNA-27a mediated targeting of ZBTB10. Betulinic acid significantly reduced the quantities of Sp1, Sp3 and Sp4 in the tissues of the tumors derived from RKO cells [70].

Interestingly, quantities of Sp1, Sp3 and Sp4 were found to be suppressed in ZBTB4-overexpressing cancer cells. Sp1 binding sites have previously been identified in the promoter region of EZH2. Transfection of MCF-7 and MDA-MB-231 cancer cells with miRNA-20a, miRNA-106a and miRNA-106b antagomirs led to evident suppression in the levels of Sp1, Sp3, Sp4 and EZH2, but, simultaneously, there was an increase in the levels of ZBTB4. Betulinic acid downregulated Sp1, Sp3, Sp4 and EZH2 in tumor tissues and this was accompanied by simultaneous decrease in the levels of miR20a, miR-106a and miR-106b [71].

miR-22-3p inhibitors caused suppression of the apoptosis of HCC cells, while miR-22-3p mimics promoted the apoptotic cell death. MALAT1 was reduced by betulinic acid in HCC cells as well as in the tumor tissues of xenografted animal models. Betulinic acid increased the apoptosis in miR-22-3p mimics-transfected cells as compared to miRNA-22-3p inhibitors-transfected cancer cells. MALAT1 served as a sponge for miRNA-22-3p and blocked miR-22-3p-induced targeting of XIAP and survivin [72].

It seems relevant to mention that future research methodologies must converge on the identification of tumor suppressor and oncogenic lncRNAs likely to be regulated by betulinic acid. Therefore, unraveling of the mechanistic regulation of lncRNAs and circular RNAs by betulinic acid will provide concrete evidence about tumor inhibitory roles of betulinic acid during carcinogenesis and metastasis.

## 9. Animal Models: Pharmacological Testing of Betulinic Acid in the Inhibition of Carcinogenesis and Metastasis

AMPK-mTOR-ULK1 cascade centrally regulates betulinic acid-mediated autophagy-dependent apoptotic death. Importantly, levels of cleaved caspase 3, LC3B-II and p-AMPK were found to be enhanced in the tumor tissues of mice subcutaneously inoculated with T24 cells [73].

Betulinic acid restored the sensitivity of K562R cells to imatinib through inhibition of HDAC3. Specifically, betulinic acid promoted the degradation of HDAC3 and promoted an increase in acetylated histones H3 and H4. Imatinib and betulinic acid combinatorially reduced tumor growth in mice subcutaneously inoculated with K562R cells [74].

Betulinic acid efficiently triggered DNA damage (γH2AX) and apoptosis (caspase-3 and p53 phosphorylation) in temozolomide-sensitive and temozolomide-resistant glioblastoma cells. Betulinic acid reduced tumor size and prolonged survival in orthotopic GBM animal models [75].

IGFBP5 physically interacted with RASSF1C and impaired RASSF1C-mediated increase in the levels of PIWIL1 mRNA and protein levels in lung cancer cells. Betulinic acid-mediated anti-proliferative effects were impaired in RASSF1C-overexpressing A549 and NCI-H1299 cells. Moreover, betulinic acid caused downregulation in the levels of PIWIL1 in A549 and H1299 cells. Co-expression of RASSF1C-IGFBP5 made cancer cells highly sensitive to betulinic acid [76].

Betulinic acid effectively reduced GLI1, GLI2 and PTCH1 in RMS-13 cells. Intraperitoneal injections of betulinic acid led to significant retardation in the growth of RMS-13 xenografts. Betulinic acid caused a more regional expression of GLI1 leaving large tumor areas unstained compared to homogeneously stained regions of GLI1 in tumor tissues of untreated mice [77].

For the maintenance of structural architecture, bone homeostasis is mechanistically orchestrated through the push and pull between bone resorptive functions by osteoclasts and bone forming ability by osteoblasts. Essentially, this diametrically opposed process is mediated via receptor activator of NF-қB (RANK)/RANK ligand (RANKL) system. Mostly, osteolytic factors secreted by cancer cells are widely acclaimed to trigger osteoclastic differentiation and activities through RANKL expression by osteoblasts/stromal cells. Importantly, betulinic acid prevented osteoclast-mediated bone resorptive properties by interruption of RANKL-mediated osteoclastogenesis and reduced the secretions of cathepsin K and MMPs from mature osteoclasts. Therefore, intratibial injections of MDA-MB-231 cancer cells induced bone lesions in nude mice. Betulinic acid downregulated the expression of PTHrP in tumor tissues in tibial bone marrow in rodent models. Consequently, administration of betulinic acid through oral route effectively suppressed breast cancer cell-mediated bone loss [78].

**Figure 4:**
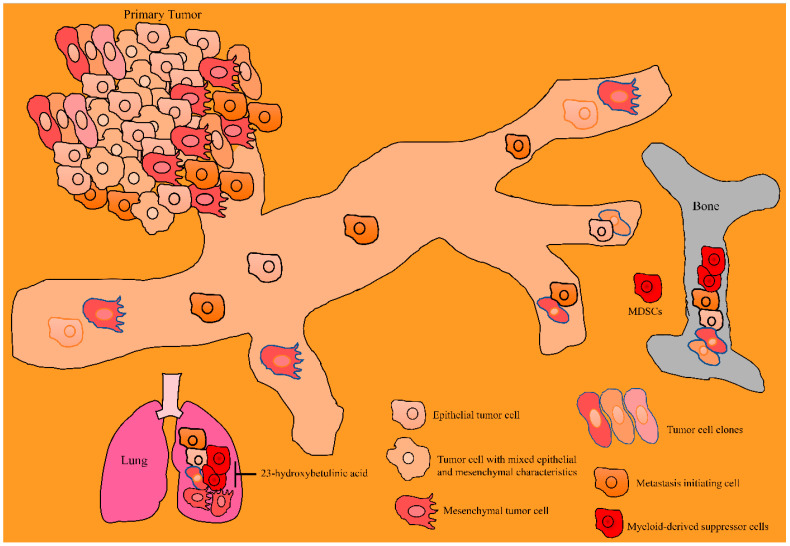
Betulinic acid mediated inhibition of invasion and colonization of cancer cells to the distantly located organs. Cancer cells from the primary tumors are metastatically competent and invade bones and lungs for colonization. Betulinic acid attenuated the invasion and colonization of cancer cells to the bones and lungs. Abbreviations: Myeloid-derived suppressor cells (MDSC), Matrix metalloproteinases (MMPs), Tissue Inhibitor of Metalloproteinase (TIMP).

Modified derivatives of betulinic acid have been shown to be effective against cancers. SH-479 (modified derivative of betulinic acid) reduced the capacities of MDA-MB-231 cancer cells to induce differentiation of osteoclasts. SH-479 significantly increased CD3 + CD4 + T lymphocytes and concordantly reduced MDSCs in the bone marrow microenvironment (Figure 4) [79].

SYK023, another betulinic acid derivative was found to be effective against animal models of lung cancer driven by EGFRL858R or KrasG12D. SYK023 robustly blocked doxycycline-induced lung cancer formation in EGFRL858R mice. Histological evaluation of EGFRL858R or KrasG12D mice clearly provided evidence of shrinkage of tumor formation in mice treated with SYK023. Synaptopodin, an actin-binding protein has been found to be frequently overexpressed in lung cancer. SYPD was reduced in the lung tissues of KrasG12D mice treated with SYK023 [80].

Sp1 has been noted to transcriptionally upregulate lamin B1 in pancreatic cancer. Lamin B1 overexpression contributed to invasion of pancreatic cancer cells. There was a considerable impairment in the invasive potential of lamin B1-silenced AsPC-1 and PANC-1 cancer cells. Importantly, lamin B1 inhibition significantly attenuated tumor growth of pancreatic cancer cells. Betulinic acid markedly induced shrinkage of tumors derived from AsPC-1 and PANC-1 cancer cells [81].

Vincristine and betulinic acid significantly reduced the pulmonary metastatic nodules in mice injected with B16F10 melanoma cells [82].

Betulinic acid suppressed the levels of MMP-2 and MMP-9, but stimulated the levels of TIMP-2 in HCT116 cells. Tumor growth was significantly suppressed by intraperitoneally administered betulinic acid in a xenograft model of HCT-116 [83].

It has also been reported that betulinic acid exerted inhibitory effects on MMP-2 and MMP-9 in HepG2 cells. Betulinic acid efficiently suppressed multiple large metastatic nodules on the surface of the lungs in mice injected with HepG2 cells [84].

Betulinic acid significantly reduced Ki67-positive and MMP-9-positive cells in the tumor tissues of mice inoculated with 786-O cancer cells [85].

23-hydroxybetulinic acid remarkably reduced CD11b + Gr1+ myeloid-derived suppressor cells in the tumor microenvironments (Figure 4) [86].

## 10. Concluding Remarks

Preclinical and clinical studies have revealed spatial and temporal intra-tumor heterogeneity in cancers and phenotypically divergent characteristics reflected by cancer cells emphasized on presence of drug resistance, loss of apoptosis and metastasizing potential of cancer cells. Research over the years has sequentially provided deeper comprehension of the cell signaling pathways and use of newer technologies including massively parallel sequencing has provided in-depth analysis regarding origin and evolution of tumors, occurrence of new mutations and underlying mechanisms for resistance against wide ranging molecular therapeutics. In this mini-review, we have gathered scattered pieces of an incomplete jig-saw puzzle related to pharmacologically significant role of betulinic acid in cancer chemoprevention. We have exclusively discussed the regulatory roles of betulinic acid from a translational perspective, focusing on recent insights from cell culture studies and animal model studies. However, there are different pathways which still demand extensive research in the context of betulinic acid mediated cancer chemoprevention. There is a need to drill down deep into the mechanisms associated with regulation of Notch and Hippo pathways. Although certain clues have emerged related to regulation of SHH/GLI and Wnt/β-catenin by betulinic acid, it needs to be studied in detail. Essentially, betulinic acid mediated regulation of oncogenic signaling pathways needs to be complemented with more empirical and systems-wide research in the future.

## Data Availability

Not applicable.
